# INFORMAL CAREGIVING BURNOUT AMONG THE SANDWICH GENERATION

**DOI:** 10.1093/geroni/igac059.3115

**Published:** 2022-12-20

**Authors:** Erika Fenstermacher, Montgomery Owsiany, Barry Edelstein

**Affiliations:** West Virginia University, Morgantown, West Virginia, United States; West Virginia University, Morgantown, West Virginia, United States; West Virginia University, Morgantown, West Virginia, United States

## Abstract

Twenty-nine percent of U.S. adults care for children. Of those adults, 12% are multigenerational caregivers who also provide unpaid care for one or more adults. Many multigenerational caregivers are considered members of the “sandwich generation,” which is a term for multigenerational caregivers who provide care, financial support, and emotional support for both their children and parents. Approximately 71% of this generation is between the ages of 40 and 59, and approximately 10% are 60 or older. The sandwich generation is largely understudied and presents challenges including informal caregiving burnout and depression. Informal caregiving burnout is a syndrome that results from the stress of providing care. Researchers have not yet investigated burnout in the sandwich generation. The present study examined how sandwich generation caregivers differed from caregivers of children and caregivers of parents regarding depression and burnout. Two measures of burnout were used (non-caregiving burnout = degree of physical and psychological fatigue and exhaustion; informal caregiving burnout = degree of physical and psychological fatigue and exhaustion related to caregiving). We found that sandwich generation caregivers and caregivers of parents scored significantly higher than caregivers of children on informal caregiving burnout. Caregiving burnout and non-caregiving burnout were significantly correlated with depression (r = .496, p < .001 and r = .773, p < .001, respectively). Burnout is higher in sandwich generation caregivers and those who care for parents than burnout among those who care only for children. This study is unique in its investigation of informal caregiving burnout among the sandwich generation.

